# Inhibition of Semicarbazide-sensitive Amine Oxidase Reduces Atherosclerosis in Cholesterol-fed New Zealand White Rabbits

**DOI:** 10.1038/s41598-018-27551-6

**Published:** 2018-06-18

**Authors:** Shu-Huei Wang, Tse-Ya Yu, Chi-Sheng Hung, Chung-Yi Yang, Mao-Shin Lin, Chien-Yin Su, Yuh-Lien Chen, Hsien-Li Kao, Lee-Ming Chuang, Feng-Chiao Tsai, Hung-Yuan Li

**Affiliations:** 1 Department of Anatomy and Cell Biology, National Taiwan University, Taipei, Taiwan; 2 Health Management Center, Far Eastern Memorial Hospital, New Taipei City, Taiwan; 3 Department of Internal Medicine, National Taiwan University Hospital, Taipei, Taiwan; 4 Medical Imaging, National Taiwan University Hospital, Taipei, Taiwan; 5 Department of Medical Imaging, E-Da Hospital, I-Shou University, Kaohsiung, Taiwan; 6 Department of Pharmacology, College of Medicine, National Taiwan University, Taipei, Taiwan

## Abstract

Inflammation, oxidative stress, and the formation of advanced glycated end-products (AGEs) are important components of atherosclerosis. Vascular adhesion protein-1 (VAP-1) participates in inflammation. Its enzymatic activity, semicarbazide-sensitive amine oxidase (SSAO), can catalyze oxidative deamination reactions to produce hydrogen peroxide and aldehydes, leading to the subsequent generation of AGEs. This study aimed to investigate the effect of VAP-1/SSAO inhibition on atherosclerosis. In our study, immunohistochemical staining showed that atherosclerotic plaques displayed higher VAP-1 expression than normal arterial walls in apolipoprotein E-deficient mice, cholesterol-fed New Zealand White rabbits and humans. In cholesterol-fed rabbits, VAP-1 was expressed on endothelial cells and smooth muscle cells in the thickened intima of the aorta. Treatment with PXS-4728A, a selective VAP-1/SSAO inhibitor, in cholesterol-fed rabbits significantly decreased SSAO-specific hydrogen peroxide generation in the aorta and reduced atherosclerotic plaques. VAP-1/SSAO inhibition also lowered blood low-density lipoprotein cholesterol, reduced the expression of adhesion molecules and inflammatory cytokines, suppressed recruitment and activation of macrophages, and decreased migration and proliferation of SMC. In conclusion, VAP-1/SSAO inhibition reduces atherosclerosis and may act through suppression of several important mechanisms for atherosclerosis.

## Introduction

Atherosclerosis is an inflammatory disease of the arterial wall^1^, and its complications remain the principal cause of death in developed countries^2^. The lesions of atherosclerosis represent a series of highly specific cellular and molecular responses^3,4^. It is well recognized that oxidative stress and inflammation play important roles in the evolution of atherosclerosis^4,5^. Therefore, agents which reduce oxidative stress and inflammation in the arterial wall are potential drug candidates for atherosclerosis.

Vascular adhesion protein-1 (VAP-1) is an adhesion molecule that is strongly expressed in endothelial cells, smooth muscle cells (SMC), and adipocytes^6,7^. Endothelial VAP-1 is involved in leukocyte rolling, adhesion, and transmigration into inflammatory sites^8^. It also possesses enzymatic function, semicarbazide-sensitive amine oxidase (SSAO), which catalyzes the oxidative deamination of primary amines (e.g., methylamine, aminoacetone) into aldehydes (formaldehyde, methylglyoxal), hydrogen peroxide, and ammonia^9,10^. Hydrogen peroxide and ammonia are cytotoxic to vascular cells at high concentrations^11^. As a source of ROS, hydrogen peroxide can modify low-density lipoprotein (LDL) in the arterial wall. The aldehydes can induce endothelial injury through the production of advanced glycation end-products (AGEs) and the cross-linking of proteins to each other^12,13^. These effects can result in the development of atherosclerosis. To support this finding, mice fed with high-fat diet coupled with chronic administration of methylamine resulted in increased atheroma area^14^. In humans, we have demonstrated that serum VAP-1 is a source of systemic ROS and AGEs. It is associated with carotid intima-medial thickness, an index for atherosclerosis^15^. Moreover, we have also shown that serum VAP-1 can predict 10-year cardiovascular mortality in subjects with type 2 diabetes^16^. Taken together, these findings suggest that VAP-1/SSAO is involved in the development of atherosclerosis and is a novel target to reduce atherosclerosis.

So far, literature regarding the effects of VAP-1/SSAO inhibition on atherosclerosis is limited, and the results remain inconsistent. Two studies using semicarbazide to abrogate VAP-1/SSAO activity in LDL receptor knockout mice have demonstrated that VAP-1/SSAO inactivation may aggravate atherosclerotic development through induction of SMC phenotypic switching^17^, whereas it also exerts an anti-atherogenic effect by limiting leukocyte infiltration^18^. Since semicarbazide can also act on enzymes other than VAP-1/SSAO involved in atherogenesis^19–24^, off-target effects may explain the conflicting results. On the other hand, another study has not found significant difference in plaque size after 4-week treatment with small molecule VAP-1 inhibitor (LJP1586) compared with placebo group^25^. However, a 4-week treatment length may not be long enough to observe regression of atherosclerosis. Therefore, the treatment efficacy may be underestimated.

PXS-4728A is a potent, oral active low molecular weight inhibitor of VAP-1/SSAO. It has more than 500-fold selectivity for VAP-1/SSAO over all the related amine oxidases and does not have off-target effects on over 100 different macromolecular targets^26^. PXS-4728A can inhibit neutrophil rolling and tethering, as well as diminishing inflammation. It has been studied in the treatment for chronic obstructive pulmonary disease^27^ and nonalcoholic steatohepatitis^28^. However, its effect on atherosclerosis remains unknown. Therefore, the aim of the present study is to investigate whether a selective VAP-1/SSAO inhibitor (PXS-4728A) can reduce atherosclerosis in cholesterol-fed New Zealand White rabbits and to explore the potential molecular mechanisms.

## Results

### Expression of VAP-1 in atherosclerotic lesions in apolipoprotein E (ApoE)-deficient mice, cholesterol-fed rabbits, and humans

**Figure 1:**
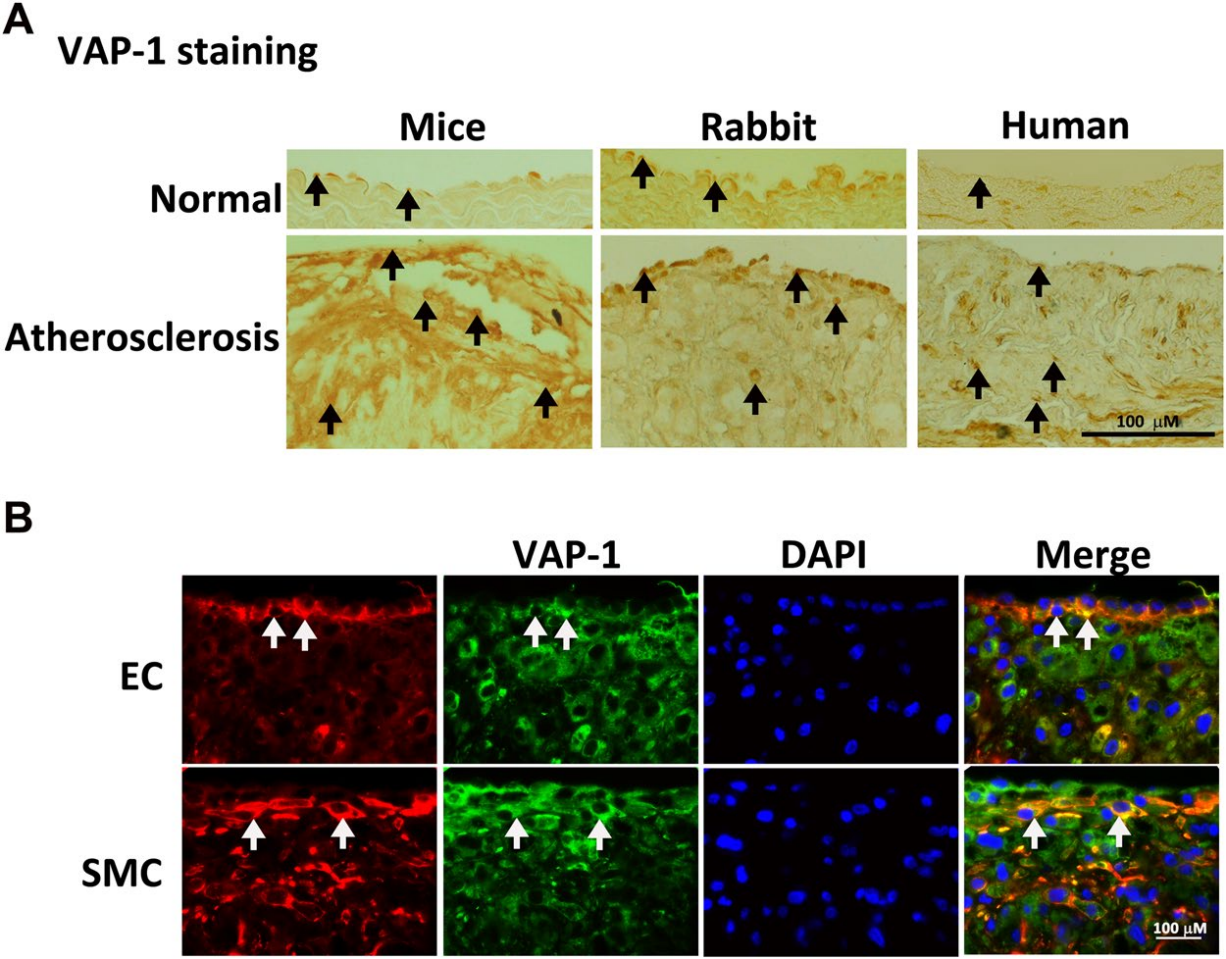
The distribution of vascular adhesion protein-1 (VAP-1) in the aorta. (**A**) Stronger VAP-1 staining (black arrows) is seen in atherosclerotic arterial walls than in normal parts in apolipoprotein-E deficient mice, rabbits, and humans. (**B**) Strong VAP-1 staining (white arrows) is seen in the markedly thickened intima of cholesterol-fed rabbits and is closely co-localized with endothelial cells (EC) and smooth muscle cells (SMC). The scale bar = 100 μm.

To examine VAP-1 expression during atherosclerosis in mice, rabbits and humans, immunohistochemical staining was performed with antibodies against VAP-1 (Fig. 1A). In the normal aorta, VAP-1 staining was seen only in a few parts of the luminal surface and tunica media (Fig. 1A). Compared to the normal aorta, VAP-1 expression was significantly stronger in the atherosclerotic lesions in thoracic aorta of mice and rabbits and in the carotid artery of humans. To examine the cellular localization of VAP-1 during the formation of atherosclerosis in rabbits, immunohistochemical staining with antibodies against VAP-1, endothelial cells (CD31) or SMC (α-actin) were carried out in the rabbit aorta sections (Fig. 1B). VAP-1 staining overlaid with endothelial cells and SMC (Fig. 1B). These data indicated that endothelial cells and SMC can express VAP-1.

### PXS-4728A inhibits SSAO activity and H_2_O_2_ generation in cholesterol-fed rabbits

**Figure 2:**
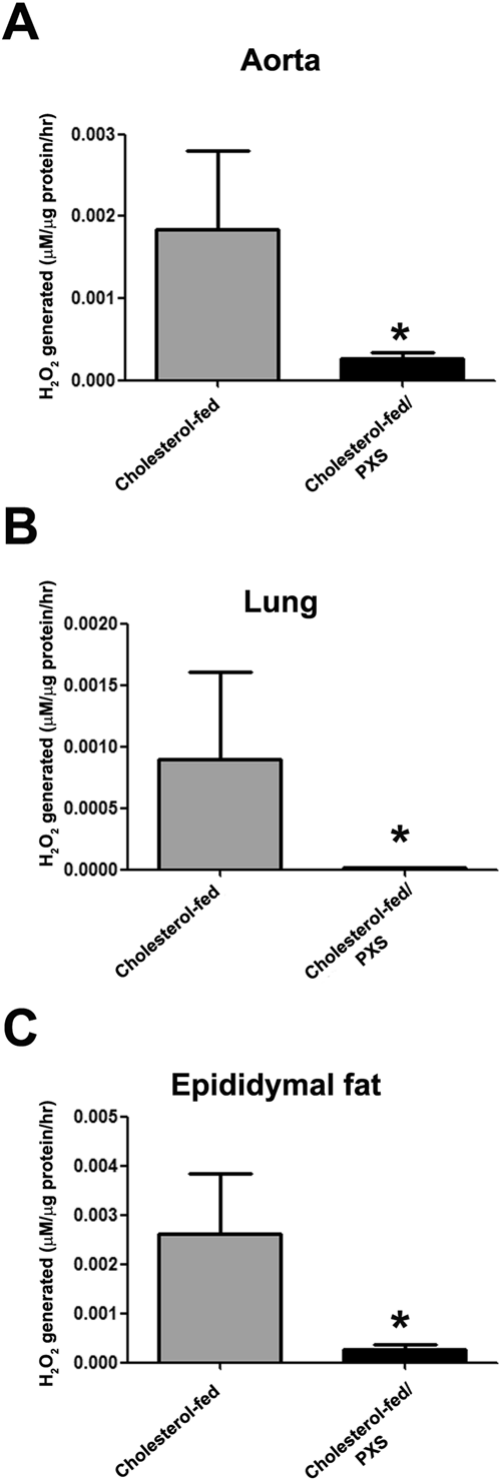
Effect of PXS-4728A on semicarbazide-sensitive amine oxidase (SSAO) activity in (**A**) thoracic aorta, (**B**) lung, and (**C**) epididymal fat. SSAO activity is expressed as the SSAO-specific production rate of H_2_O_2_. The values are the mean ± SEM (N = 5–6 in each group). *p < 0.05 compared to the cholesterol-fed group.

To detect the effects of PXS-4728A treatment on SSAO activity, we measured H_2_O_2_ production rate from SSAO in cholesterol-fed rabbits. To find an appropriate dose of PXS-4728A on SSAO inhibition, we measured the SSAO activity of abdominal fat after dosing PXS-4728A at 1 mg/kg or 10 mg/kg for 4 consecutive days. Compared with the control, PXS-4728A at 1 mg/kg showed 80% inhibition of SSAO activity, whereas PXS-4728A at 10 mg/kg demonstrated greater than 90% inhibition of SSAO activity. Therefore, PXS-4728A at 10 mg/kg was used in the following experiment. In Fig. 2, PXS-4728A at 10 mg/kg significantly inhibited SSAO activity and SSAO-specific H_2_O_2_ production in thoracic aortic tissues, lung, and epididymal fat.

### Effects of SSAO inhibition by PXS-4728A on body weight and serum biochemical parameters of cholesterol-fed rabbits

As shown in Supplementary Table S1, there was no significant difference among the three groups in basal body weight, plasma total cholesterol (TC), LDL-cholesterol (LDL-C), high-density lipoprotein cholesterol (HDL-C), glucose, and triglyceride concentrations at day 0 before treatment. After 12 weeks (Table 1), body weight, plasma TC, LDL-C, HDL-C, and glucose concentrations were significantly increased in the cholesterol-fed rabbits as compared with the control group (p < 0.05). The elevation of body weight, plasma TC, LDL-C, and glucose concentrations were significantly reduced in cholesterol-fed rabbits treated with PXS-4728A (p < 0.05). There was no difference in plasma creatinine among the three groups. Besides, plasma alanine transaminase (ALT) and aspartate aminotransferase (AST) levels were higher in the rabbits fed a high-cholesterol diet than those in the control group. In cholesterol-fed/PXS-4728A group, AST levels were significantly decreased compared with the cholesterol-fed group. However, ALT levels showed a trend of reduction, which did not reach significance (Table 1).

**Table 1:** Fasting plasma parameters of rabbits after 12 weeks of treatment.

	Control (n = 4)	Cholesterol Diet (n = 6)	Cholesterol Diet /PXS-4728A (n = 5)
Body weight (kg)	3.09 ± 0.15	3.58 ± 0.05^*^	3.28 ± 0.11^**†**^
TC (mg/dl)	109.9 ± 6.34	695.9 ± 66.12^*^	508.4 ± 17^**†**^
LDL-C (mg/dl)	27.62 ± 7.23	576.8 ± 61.9^*^	403.2 ± 13.56^**†**^
HDL-C (mg/dl)	82.29 ± 1.10	119.1 ± 11.25^*^	105.2 ± 9.26
Glucose (mg/dl)	155.7 ± 5.33	258.7 ± 31.4^*^	195.3 ± 13.53^**†**^
Triglyceride (mg/dl)	122.1 ± 16.01	367.6 ± 125.6	231.9 ± 16.85
Creatinine (mg/dl)	1.3 ± 0.33	1.77 ± 0.17	1.94 ± 0.08
ALT (U/I)	133.1 ± 8.83	356.2 ± 32.3^*^	285.3 ± 36.76
AST (U/I)	34.05 ± 4.65	502.8 ± 79.14^*^	209.2 ± 19.45^**†**^

Values are mean ± S.E.M.

Abbreviations: TC, total cholesterol; LDL-C, low-density lipoprotein cholesterol; HDL-C, high-density lipoprotein cholesterol; ALT, alanine transaminase; AST, aspartate aminotransferase.

*p < 0.05 compared with the control group. ^†^p < 0.05 compared with the cholesterol diet group.

### SSAO inhibition by PXS-4728A reduces atherosclerosis in cholesterol-fed rabbits

**Figure 3:**
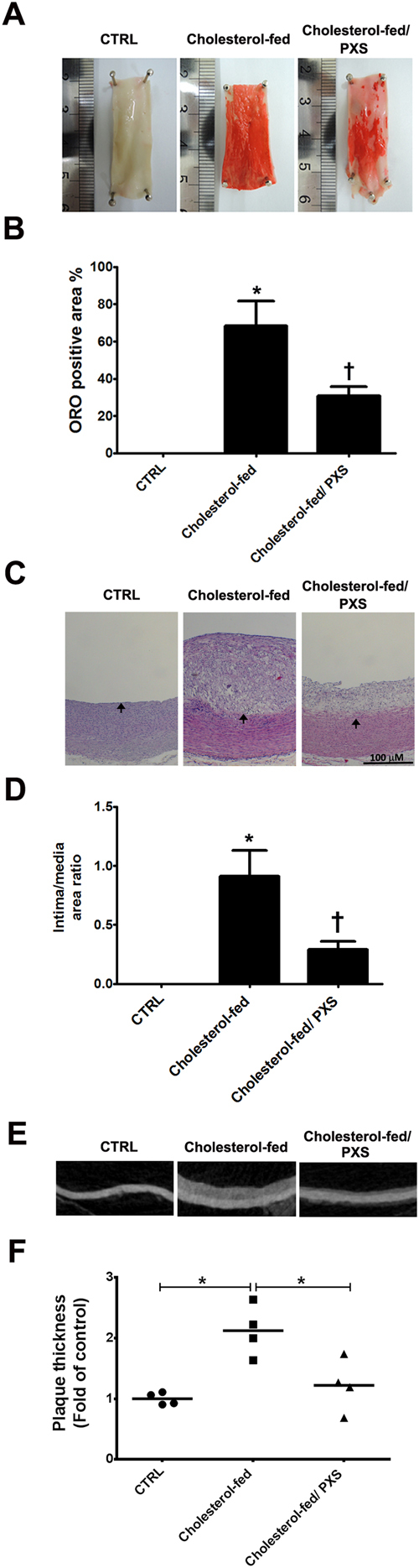
Effects of semicarbazide-sensitive amine oxidase (SSAO) inhibition by PXS-4728A on atherosclerotic lesions. (**A**) Representative photographs showing oil red O-stained atherosclerotic plaques of the aorta in different groups. (**B**) Quantification of oil red O-stained atherosclerotic plaques in different groups. (**C**) Representative photographs showing Hematoxylin and Eosin staining of atherosclerotic plaques of the aorta in different groups. (**D**) Quantification of the ratio of intima/media area in different groups. (**E**) Representative photographs showing atherosclerotic plaques of the aorta by micro-computed tomography (micro-CT) in different groups. (**F**) Quantification of atherosclerotic plaques by micro-CT in different groups. In B, D and F, the data are expressed as a ratio and calculated as the mean ± SEM (n = 4–6 in each group); *p < 0.05 compared to the control group (CTRL). ^†^p < 0.05 compared to the cholesterol-fed group.

The effects of PXS-4728A on the atherosclerotic plaque area were quantified by histomorphometric analysis of the thoracic aortic cross sections in the rabbits 12 weeks after cholesterol diet treatment. The atherosclerotic plaque area was determined by oil red O staining analysis. Compared with the cholesterol-fed group, SSAO inhibition by PXS-4728A reduced atherosclerotic plaque area by 37.59% (68.31% ± 13.34% in the cholesterol-fed group versus 30.72% ± 5.12% in the cholesterol-fed/PXS-4728A group, p < 0.05, Fig. 3A,B).

The intima/media area ratio and plaque thickness of the thoracic aortas were significantly increased in the cholesterol-fed rabbits than in the control group by histological staining analysis (Fig. 3C,D) and by Micro-computed tomography (Micro-CT) (Fig. 3E,F). In contrast, compared with that in the cholesterol-fed group, SSAO inhibition by PXS-4728A resulted in a significant decrease in the intima/media area ratio (0.91 ± 0.22 in the cholesterol-fed group versus 0.29 ± 0.07 in the cholesterol-fed/PXS-4728A group, p < 0.05, Fig. 3C,D) and thickness of the aortic wall (2.12 ± 0.21 in the cholesterol-fed group versus 1.22 ± 0.22 in the cholesterol-fed/PXS-4728A group, p < 0.05, Fig. 3E,F). Altogether, the results show that SSAO inhibition by PXS-4728A reduces atherosclerosis in cholesterol-fed rabbits.

### SSAO inhibition by PXS-4728A reduces the expression of adhesion molecules, inflammatory cytokines, macrophage recruitment, and macrophage activation in atherosclerotic plaques

**Figure 4:**
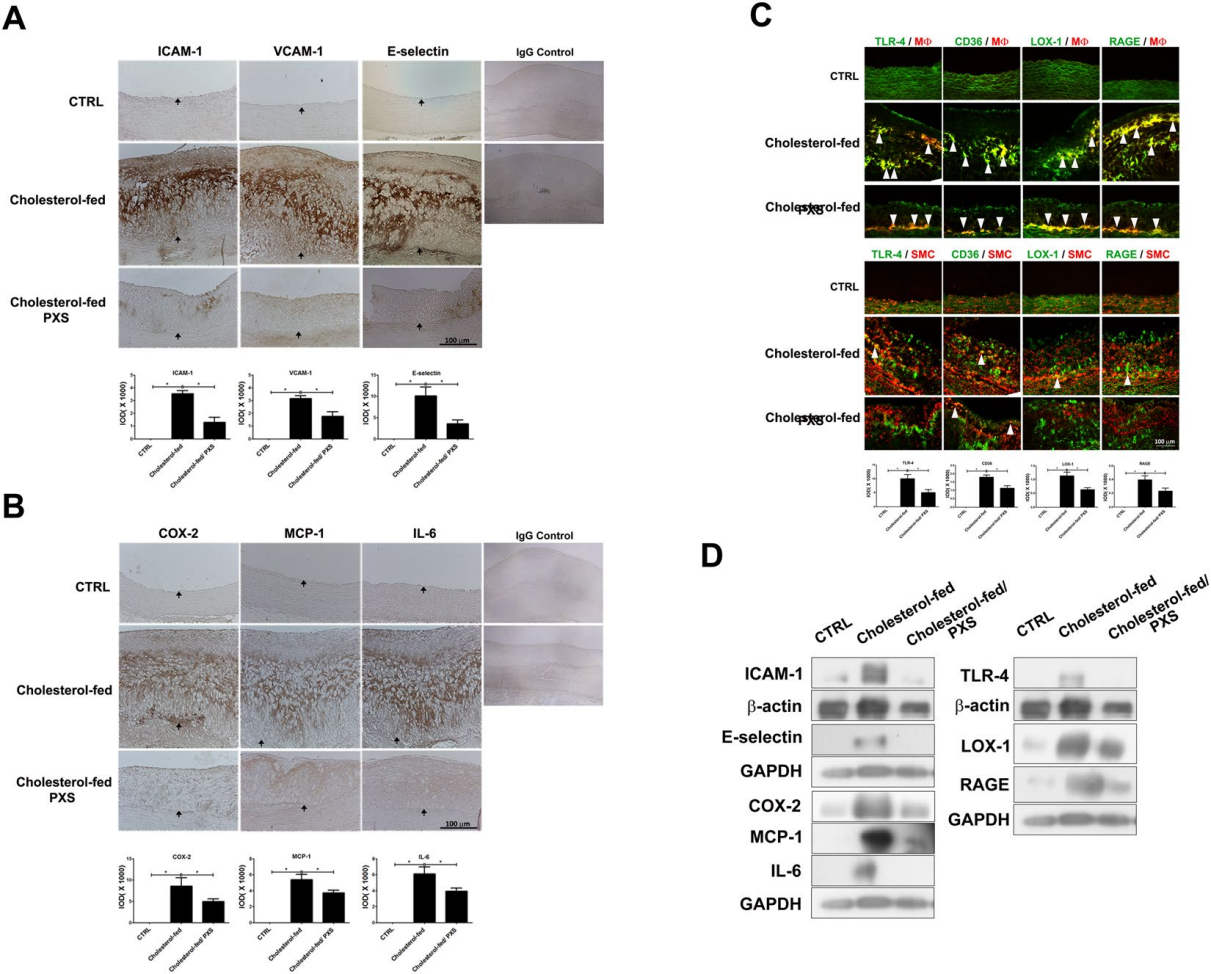
Effects of semicarbazide-sensitive amine oxidase (SSAO) inhibition by PXS-4728A on the adhesion, inflammation, and macrophages activation in atherosclerotic plaques of thoracic aorta in rabbits in different groups. (**A**) Expression of adhesion molecules in the atherosclerotic plaques in different groups. Immunohistochemical staining for intercellular adhesion molecule-1 (ICAM-1), vascular cell adhesion molecule-1 (VCAM-1), and E-selectin expression are shown. (**B**) Expression of inflammatory cytokines including cyclooxygenase-2 (COX-2), monocyte chemoattractant protein-1 (MCP-1), and interleukin-6 (IL-6) by immunohistochemical staining in different groups. (**C**) Expression of markers for macrophage activation and recruitment in different groups. Immunohistochemical staining for Toll-like receptor -4 (TLR-4), CD36, lectin-like oxidized low-density lipoprotein receptor-1 (LOX-1), receptor for advanced glycation end-product (RAGE), macrophage (Mϕ) and smooth muscle cells (SMC) expression are shown. Strong TLR-4, CD36, LOX-1, and RAGE staining (white arrowheads) is seen in the markedly thickened intima of cholesterol-fed rabbits and is closely co-localized with macrophages (Mϕ) and SMC. Expression of adhesion molecules, cytokines, and macrophage activation markers are higher in cholesterol-fed group than that in control (CTRL) group and cholesterol-fed/PXS-4728A group. The internal elastic lamina is indicated by the arrows. The scale bar = 100 μm. Quantification of IHC staining-positive areas in atherosclerotic plaques of thoracic aorta were shown on the lower panel of indicated antibodies (N = 3–4 in each group). Histograms of their integrated optical density (IOD) value were measured by Image-Pro Plus software. The values are expressed as mean ± SEM. (*P < 0.05). (D) Expression levels of the markers of adhesion, inflammation, and macrophages activation in rabbit aorta are detected by Western blot analysis in different groups. The cropped blots are used in the figure, and the full-length blots are presented in Supplementary Figure S5.

To test the anti-inflammation effects of the PXS-4728A, immunohistochemical staining and Western blot analyses with antibodies against adhesion and inflammatory molecules were carried out on thoracic aorta sections (Fig. 4 and Supplementary Figure S2). The expression of adhesion molecules, including intercellular adhesion molecule-1 (ICAM-1), vascular cell adhesion molecule-1 (VCAM-1), and E-selectin, were higher in thoracic aorta of rabbits in the cholesterol-fed group than that in the normal control group (Fig. 4A). Compared with the cholesterol-fed group, SSAO inhibition by PXS-4728A significantly decreased the expression of these adhesion molecules (all p < 0.05) (Fig. 4A,D). Similarly, expression of pro-inflammatory molecules (COX-2 and IL-6) and monocyte chemoattractant protein-1 (MCP-1) was higher in the cholesterol-fed group and was reduced in the cholesterol-fed/PXS group (all p < 0.05) (Fig. 4B,D). During the development of atherosclerosis, macrophages are recruited to plaques, where they become activated by AGEs and oxidized LDL^29^. Activated macrophages become foam cells, which consequently propagate inflammation and progress to atherosclerosis^30,31^. To test the effect of SSAO inhibition by PXS-4728A on AGEs-LDL-activated macrophages in the atherosclerotic plaques, immunohistochemical staining and Western blot analyses with antibodies against the receptor for AGEs (RAGE), lectin-like oxidized LDL receptor-1 (LOX-1), CD36, and Toll-like receptor-4 (TLR-4) were performed in rabbit thoracic aorta sections. Since vascular smooth muscle cells may have phenotypic switching to macrophage-like cells and foam cells^32^, we confirmed this by performing double staining for markers of SMC and macrophages (Supplementary Figure S3). Therefore, these macrophage activation markers were double stained with macrophage or smooth muscle cell markers. As shown in Fig. 4C,D and Supplementary Figure S4, the cholesterol-enriched diet resulted in a significant increase in the presence of macrophages, and the expression of RAGE, LOX-1, TLR-4, and CD36 in the atherosclerotic plaques, but these increasing effects were markedly decreased in cholesterol-fed rabbits administered PXS-4728A (all p < 0.05). A strong TLR-4, CD36, LOX-1 and RAGE staining was seen in the markedly thickened intima of cholesterol-fed rabbits and was closely co-localized with both the macrophage marker and the smooth muscle cell marker, suggesting that PXS-4728A reduced macrophage activation in both monocyte-derived and SMC-derived macrophages.

### SSAO inhibition by PXS-4728A reduces the expression of matrix metalloproteinase-9 (MMP-9), SMC, and PCNA in atherosclerotic plaques

**Figure 5:**
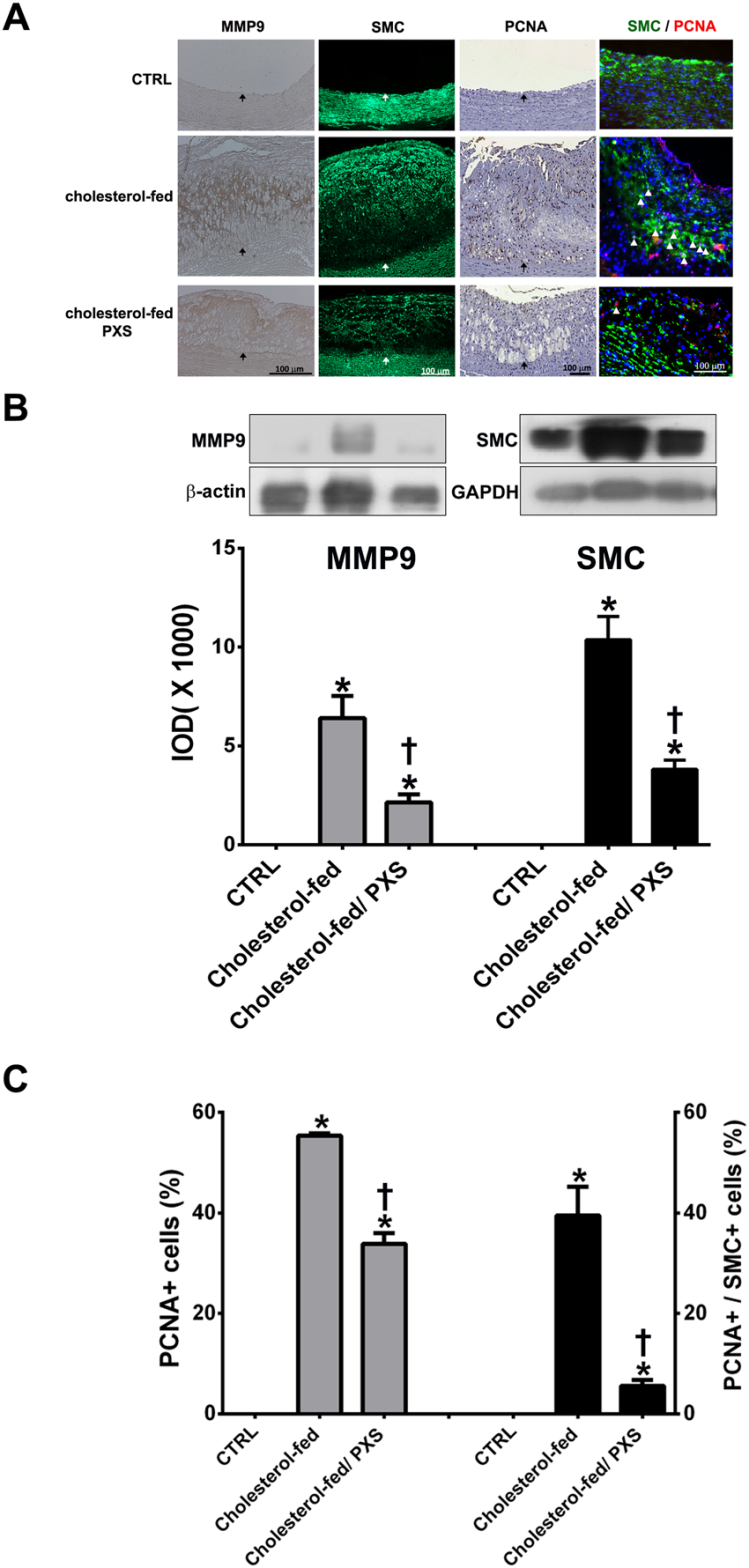
The immunohistochemical analysis for the matrix metalloproteinase-9 (MMP-9) expressions, smooth muscle cells (SMC), proliferative cell nuclear antigen (PCNA) (brown color) in aortas of rabbits in different groups. (**A**) Compared to rabbits with normal diet (CTRL), cholesterol-fed rabbits had markedly thickened intima with increased MMP-9, SMC, and PCNA staining. The immunopositive staining of MMP-9, SMC, PCNA, and co-localization between PCNA and SMC expressions in cholesterol-fed rabbits with PXS-4728A treatment (Cholesterol-fed/PXS-4728A) were weaker and lower than that in cholesterol-fed rabbits. The internal elastic lamina is indicated by the arrows. The scale bar = 100 μm. (**B**) The integral optical density (IOD) of MMP-9 immunostaining intensity quantified by Image-Pro Plus software (N = 3–4 in each group). (**C**) Percentage of proliferating cells (PCNA-positive) determined by dividing the number of PCNA-positive cells by the number of total cells per section (N = 3–4 in each group). The values are expressed as mean ± SEM. *p < 0.05 compared to control group (CTRL). ^†^p < 0.05 compared to Cholesterol-fed group.

The proliferation and migration of vascular SMC play an important role in the progression of atherosclerosis^33^. SMC migrate from the tunica media into the intima via degradation of the extracellular matrix mediated by MMP-9 and other proteinases^34^. Immunohistochemical staining and Western blot analyses of MMP-9 and the SMC marker, α-actin, revealed stronger MMP-9 expression and more α-actin-positive cells in the thickened intima of the cholesterol-fed group (Fig. 5A,B, p < 0.05 and Fig. 4D). These effects were significantly reduced by PXS-4728A treatment. To detect cell proliferation in the atherosclerotic plaque, PCNA staining was used. As shown in Fig. 5A,C, the number of PCNA-positive cells and PCNA-positive SMC in the thickened plaques was higher in the cholesterol-fed group than that in the cholesterol-fed/PXS-4728A group. Taken together, these findings suggest that SSAO inhibition by PXS-4728A reduces the migration and proliferation of SMC, as well as other cells involved in the development of atherosclerosis.

## Discussion

In the present study, we have demonstrated that VAP-1 expression is higher in atherosclerotic plaques compared to normal arterial walls. Vascular endothelial cells and SMC express VAP-1 protein. SSAO inhibition by PXS-4728A decreased H_2_O_2_ production, lowered plasma lipid levels, reduced the expression of adhesion molecules and inflammatory cytokines, suppressed recruitment and activation of macrophages, and inhibited migration and proliferation of SMC. Consequently, it results in anti-atherosclerotic effects in cholesterol-fed rabbits.

In our study, endothelial cells in atherosclerotic plaques displayed stronger VAP-1 expression than those in normal arterial wall, which is consistent with the previous report. In LDL receptor-deficient mice expressing only apolipoprotein B100 (LDLR^−/−^ApoB^100/100^), VAP-1 was expressed on the luminal surface of endothelial cells in atherosclerotic plaques, but not on endothelial cells in non-atherosclerotic part^25^. Moreover, we found that the expression of VAP-1, ICAM-1, VCAM-1, and E-selectin was higher in cholesterol-fed rabbits, and the increased expression was reduced by SSAO inhibition. In previous reports, endothelial VAP-1 has been shown to mediate leukocyte recruitment, including rolling, adhesion, and transmigration steps^8^. Compared to other classical endothelial adhesion molecules, VAP-1 can also act as an ectozyme that regulates cell recruitment through its enzymatic activity of SSAO^10^. VAP-1/SSAO inhibition effectively blocks leukocyte-endothelial interaction *in vitro* and *in vivo* and thereby attenuates leukocyte influx into inflammation sites^35–38^. Additionally, SSAO activity has been linked to up-regulation of other endothelial adhesion molecules, such as E-selectin, P-selectin, and ICAM-1, in both *in vivo* and *in vitro* experiments^39,40^. These data support our findings and indicate that endothelial VAP-1/SSAO is a key to initiate and propagate inflammation and atherosclerosis.

In the development of atherosclerosis, macrophages are important players. They secrete chemotactic proteins such as MCP-1, further recruiting more macrophages and releasing various cytokines (e.g., IL-1, IL-6, and tumor necrosis factor-α) which propagate inflammation^4^. In the present study, we found that atherosclerotic plaques in cholesterol-fed rabbits had more macrophage accumulation and higher expression of MCP-1 and IL-6 than that in the control group. SSAO inhibition can suppress macrophage recruitment and reduce the expression of MCP-1 and IL-6. These findings are in agreement with previous reports. In a rat model, SSAO inhibition suppressed the expression of MCP-1 and tumor necrosis factor-α, resulting in reduced choroidal neovascularization^41^. Similarly, decreased expression of MCP-1 and tumor necrosis factor-α by SSAO inhibition has been shown in mice with intracerebral hemorrhagic stroke^40^. Once the macrophages are recruited, they become activated. Activated macrophages express LOX-1 and CD36 to engulf oxidized LDL and become foam cells. TLR-4 expression plays an important role in activating the innate immune system^42^. The present study has demonstrated that the expression of LOX-1, CD36, and TLR-4 was higher in cholesterol-fed rabbits, and this expression was reduced by SSAO inhibition. Taken together, these findings suggest that SSAO inhibition decreases recruitment and activation of macrophage, which might be TLR-4 dependent.

The SSAO activity of VAP-1 catalyzes the oxidative deamination of endogenous amines to produce hydrogen peroxide and aldehydes, both of which may accelerate the formation of AGEs^43^. The receptor for AGEs, RAGE, is expressed on many cells, including endothelial cells, mononocytes, macrophages and SMC. The interaction of RAGE and its ligands plays a pivotal role in vascular inflammation, endothelial dysfunction, and the development of atherosclerotic plaques^44^. For example, RAGE inactivation inhibits atherosclerosis by reducing oxidized LDL-induced pro-inflammatory responses and oxidative stress in LDL receptor knockout mice^45^. In this study, SSAO inhibition decreased H_2_O_2_ production and the expression of RAGE in the vessel walls, suggesting another mechanism whereby SSAO inhibition reduces atherosclerosis.

In the present study, we found that SMC displayed strong expression of VAP-1, which is consistent with previous reports^46,47^. During the progression of SMC differentiation, the mRNA and protein expression of VAP-1 as well as SSAO activity increase significantly^48^. Mercier *et al*. have demonstrated that SSAO-deficient mice have larger arterial diameters, suggesting a role of SSAO in arterial wall remodeling^49^. Indeed, SSAO inhibition decreased MMP-9 expression in the present study. MMP-9, with its proteolytic activity, plays an important role in SMC migration and vessel remodeling^33,34^. This outcome explains why the number of SMC in atherosclerotic lesions was diminished by SSAO inhibition in this study. Additionally, the proportion of PCNA-positive cells in aorta significantly decreased with the treatment of SSAO inhibitors. These findings indicate that SSAO inhibition not only attenuates the migration of SMC but also suppresses the proliferation of SMC.

Interestingly, we found that SSAO inhibition significantly lowered plasma concentrations of fasting glucose, TC, and LDL-C in the present study. Since SSAO inhibition also reduced body weight in this study, this could be one of the mechanisms for its glucose- and lipid-lowering effects. During adipocyte differentiation, SSAO is up-regulated^50^, and substrates of SSAO can promote adipose conversion of 3T3-L1 cells^51^. In mice overexpressing human VAP-1/SSAO in endothelium, their body mass index and subcutaneous abdominal fat pad weights were increased^14^. In obese diabetic KKAy mice, SSAO inhibition also reduced body weight, which is in concordance with our findings^52^. However, VAP-1/SSAO knockout mice demonstrated a higher body weight than that of wild type mice^53^. Therefore, further studies are needed to investigate the issue, especially the different roles of SSAO in different tissues on energy homeostasis and the development of obesity. Besides, there are some human studies which suggest a link between VAP-1/SSAO, hyperlipidemia and diabetes. In subjects with diabetes mellitus, serum SSAO activity is positively correlated with hyperlipidemia^54^. Several reports^55^, including ours^56^, have shown that serum VAP-1 concentration is higher in diabetic subjects and positively associated with hemoglobin A1c. Taken together, VAP-1/SSAO might play an important role in glucose and lipid metabolism, and further studies are needed to explore these underlying mechanisms.

In the present study, VAP-1/SSAO inhibition by PXS-4728A significantly reduced atherosclerosis, which was different from previous reports in other animal models^17,18^. In LDL receptor knock-out mice, 3 or 6 weeks of SSAO inhibition by semicarbazide after feeding western diet for 6 or 9 weeks promoted the switch of SMC from a contractile phenotype to a synthetic phenotype and increased the accumulation of collagens and the thickness of cap, thereby enhancing plaque stability. However, there was no significant difference in plasma TC and plaque size between the treatment and the placebo group^18^, which was different from the findings of our study. Furthermore, if semicarbazide was administered simultaneously with western diet, it inhibited the migration of circulating monocytes into peripheral tissue and aggravated the development of atherosclerosis^17^. In comparison, another study reported a significant reduction in plaque inflammation in LDLR^−/−^ApoB^100/100^ mice by a small molecule VAP-1/SSAO inhibitor (LJP1586) for 4 week^25^. However, there was no significant change in advanced atherosclerotic plaques. Some possible reasons may underlie the discrepancies between our study and previous findings. First, semicarbazide is a non-specific VAP-1/SSAO inhibitor. It can also act on enzymes other than VAP-1/SSAO, such as lysyl oxidase (LOX) and sphigosine-1-phosphate (S1P) lyase^20^. LOX participates in extracellular matrix maturation and proliferation, as well as SMC migration. Inhibition of LOX activity could results in endothelial dysfunction and plaque progression^21^. Besides, S1P lyase is a key regulator of S1P signaling^19^ which is involved in attachment and migration of monocytes, SMC proliferation, and production of pro-inflammatory cytokines^22,23^. The precise role of S1P signaling in atherosclerosis is complex, with both pro- and anti-atherogenic actions being identified depending on cellular and animal model used^24^. This could explain why VAP-1/SSAO inhibition by semicarbazide has an inconsistent effect on atherogenesis. In contrast, we used a highly specific VAP-1/SSAO inhibitor (PXS-4728A) with more than 500-fold selectivity over all the related amine oxidases and over 100 different macromolecular targets^26^. Therefore, off-target effect other than VAP-1/SSAO inhibition is minimized in our study. Second, the other reason for discrepant results is the treatment length. Even though LJP1586 is a selective VAP-1/SSAO inhibitor, the treatment period of 4 weeks may not have been long enough to observe significant changes in plaque size. Therefore, the effect of VAP-1/SSAO inhibition on atherosclerosis may be underestimated. In addition, the animal models used and the effect of VAP-1/SSAO inhibition on plasma lipid levels were different in these studies. These could also contribute to the different results in these studies.

In conclusion, inhibition of VAP-1/SSAO reduces atherosclerosis in cholesterol-fed New Zealand White rabbits, which may act through the suppression of several important mechanisms for atherosclerosis, including reducing oxidative stress, lowering plasma lipids, decreasing endothelial cell activation, reducing recruitment and activation of macrophages, and suppressing migration and proliferation of SMC.

## Methods

### Animal groups and treatment

Adult male New Zealand White rabbits (2 ± 0.5 kg weight) and ApoE-deficient mice were placed in cages with food and water available at all times and maintained under standard conditions with ambient temperature ~25 ± 2 °C and a regular 12 h light/12 h dark cycle. All procedures were performed in accordance with the local institutional guidelines for animal care of the National Taiwan University and complied with the “Guide for the Care and Use of Laboratory Animals” NIH publication No. 86–23, revised 2011. The protocol was also approved by the National Taiwan University College of Medicine and College of Public Health Institutional Animal Care and Use Committee (IACUC NO: 20120312).

The ApoE-deficient mice were purchased from Jackson Laboratory (Bar Harbor, ME) and maintained under a C57BL/6 background. Six animals were placed on a standard commercial mouse chow diet for 6 months and then moved to a 0.15% cholesterol diet (Purina Mills, Inc., USA) for 15 weeks. C57BL/6 mice fed with the normal diet throughout the entire period were used as the controls. After 3 months on the diet, the mice were euthanized with sodium pentobarbital (120 mg/kg i.p.) and the thoracic aorta was gently dissected, followed by paraffin-embedding and cross-sectioning for immunohistochemistry.

Rabbits were purchased from the Taiwan Livestock Research Institute (Tainan, Taiwan). Animals were randomly distributed into three groups: Group I (Control), fed a standard rabbit chow diet; Group II (Cholesterol Diet), fed a 0.5% cholesterol-enriched diet for 12 weeks; and Group III (Cholesterol Diet /PXS-4728A), fed a 0.5% cholesterol-enriched diet and PXS-4728A (10 mg/kg/day, Pharmaxis) orally for 12 weeks. The body weight was measured once every 21 days to calculate the average weight of each group. Fasting blood samples were collected from the ear marginal veins one day before the experiment commencement (0 time) and at the end of the experiment (after 12 weeks) for measurement of plasma triglycerides, TC, LDL-C, HDL-C, glucose, creatinine, ALT, and AST (Randox Laboratories. Ltd., UK).

Frozen tissue section slides of human carotid arteries were purchased from the BioCat (Heidelberg, Germany).

### Micro-computed tomography

Thoracic aortas were fixed with 4% paraformaldehyde and then analyzed by micro-CT (SkyScan 1176; Bruker, Belgium). The images were collected at a resolution of 9 μm/pixel. Reconstruction of sections was carried out with scanner software (NRecon). The 3D images were obtained with the scanner software (CTvox).

### Evaluation of atherosclerotic lesions and immunohistochemical staining

For the pathomorphological examination, samples obtained from the aortic specimen were fixed in 4% paraformaldehyde solution, conducted with routine paraffin embedding and sectioned. Serial, 5 μm-thick sections were collected throughout the length of each segment. Sections of thoracic aorta stained with Hematoxylin and Eosin (H&E) stain were also analyzed for I/M ratio (I/M = the maximum intima thickness/media thickness). The analyses were performed microscopically; the images were analyzed with Image-Pro Plus. All sections were immunohistochemically stained with CD31 (endothelial marker, Abcam, Cambridge, UK), α-actin (SMC marker, GeneTex, Irvine, California), RAM-11 (macrophage marker, Dako, Carpinteria, CA), CD36 (Abcam, Cambridge, UK), E-selectin (Cell Signaling, Danvers, MA), VAP-1 (Santa Cruz, Delaware Avenue, CA), VCAM-1 (Santa Cruz, Delaware Avenue, CA), MMP-9, COX-2, ICAM-1, MCP-1, IL-6, TLR-4, LOX-1, and RAGE antibodies (all from GeneTex, Irvine, California), and PCNA (proliferation marker, Santa Cruz, Delaware Avenue, CA), and then reacted with HRP-conjugated secondary antibodies (1:6000 in 1.5% BSA; GeneTex, Irvine, California), followed by incubation with 3,30-diaminobenzidine (Vector Lab, USA), and observed by light microscopy. Immunohistochemical staining was recorded by light microscopy (LEICA) and analyzed by Image-Pro Plus (Media Cybernetics, Crofton, MA, USA). The results are expressed as the average integrated optimal density (IOD) per unit area. The IOD was calculated by measuring 6 individual high power fields (HPF), and these data were gathered to calculate the mean value and SEM. The specificity of VAP-1 antibody was demonstrated in Supplementary Figure S1. VAP-1 stains were found in adipose tissue and thoracic aorta (positive controls), but not in spleen (negative control)^57^.

### Oil red O staining

Atherosclerotic plaque development was quantified by histomorphometry of oil red O-stained thoracic aorta. Sections of thoracic aorta stained with oil red O were performed according to a standard protocol. Briefly, after being rinsed with water, the aorta was exposed to isopropanol (60%) for 2 min, followed by 1 h of staining with a solution of oil red O dissolved in 60% isopropanol. The excess stain was removed with isopropanol and water, and the aorta was photographed using a microscope equipped with a digital camera (Leica). The red-stained areas representing atherosclerotic plaques were measured using Image-Pro Plus. The results were expressed as the ratio of total plaque area over total vessel area.

### Western blot detection

The thoracic aorta was lysed with RIPA lysis buffer (Cell Signaling, Beverly, MA, USA). A total of 20 µg of protein was separated by 10–12% SDS–PAGE and transferred onto PVDF membranes (Millipore, Bedford, MA, USA). After blocking with 5% BSA at room temperature for 1 h, the membranes were incubated with primary antibodies at 4 °C overnight. The membranes were next incubated with HRP-conjugated secondary antibodies at room temperature for 1 h. Immunoreactivity was detected with ECL (GE Healthcare Bioscience, Piscataway, NJ, USA).The intensities of the bands were quantified using Gel-Pro software (Media Cybernetics, Rockville, MD, USA). β–actin antibody was used as an internal control (1:3000 in 1.5% BSA; GeneTex, Irvine, California). The intensities of the target proteins were normalized by the intensities of the internal control bands.

### SSAO activity analysis

SSAO activity was determined as a result of the turnover of hydrogen peroxide, which was measured by Amplex Red Hydrogen Peroxide kit A22188 (Molecular Probes, Eugene, OR, USA). Briefly, 100 μg of protein was incubated at room temperature with clorgyline 1 μM (monoamine oxidase-A inhibitor), pargyline 3 μM (monoamine oxidase-B inhibitor), benzylamine 2 mM (substrate of SSAO), 100 μM Amplex Red reagent, and 1 U/ml HRP, with or without semicarbazide 1 mM. Absorbance at 570 nm was measured every 5 minutes for 30 minutes. A standard curve was plotted using different solutions with known hydrogen peroxide concentrations. The production rate of hydrogen peroxide was calculated and expressed as [H_2_O_2_]/min/μg protein. SSAO activity was determined by the difference between the production rates of hydrogen peroxide with and without semicarbazide. The linearity (R^2^) of this assay is 0.9989~1.0000 and the intra- or inter-assay CV is 0.5~3.8% in our lab.

### Statistical analysis

All values are provided as the mean ± SEM. Statistical comparisons were made using the Student’s t-test and one-way ANOVA followed by Tukey’s Multiple Comparison Test. Significance was defined as p-values < 0.05.

## Electronic supplementary material

Supplementary Table and Figures
