# Supplementary material for: Inhibition of Semicarbazide-sensitive Amine Oxidase Reduces Atherosclerosis in Cholesterol-fed New Zealand White Rabbits
Source: Sci Rep. 2018 Jun 18;8:9249. doi: 10.1038/s41598-018-27551-6 (PMC6006253; doi:10.1038/s41598-018-27551-6)
Supplement: Supplementary file 1 — Supplementary Table and Figures [file 41598_2018_27551_MOESM1_ESM.pdf]

Inhibition of Semicarbazide-sensitive Amine Oxidase Reduces Atherosclerosis in Cholesterol-fed New Zealand White Rabbits

Shu-Huei Wang,* Tse-Ya Yu*, Chi-Sheng Hung, Chung-Yi Yang, Mao-Shin Lin, Chien-Yin Su, Yuh-Lien Chen, Hsien-Li Kao, Lee-Ming Chuang, Feng-Chiao Tsai†, Hung-Yuan Li†

*These two authors contributed equally to this work.

†These two authors contributed equally to this work.

Supplementary Table S1. Fasting plasma parameters of rabbits at day 0 before treatment.

	Control (n = 4)	Cholesterol Diet (n = 6)	Cholesterol Diet /PXS-4728A (n = 5)
TC (mg/dl)	104.2 ± 2.5	100.8 ± 5.3	99.9 ± 3.8
LDL-C (mg/dl)	25.9 ± 3.3	22.8 ± 4.8	18.0 ± 3.1
HDL-C (mg/dl)	78.4 ± 1.9	78.1 ± 0.7	83.2 ± 1.8
Glucose (mg/dl)	79.9 ± 2.4	83.2 ± 3.3	77.9 ± 2.9
Triglyceride (mg/dl)	163.1 ± 32.1	154.0 ± 30.4	124.4 ± 4.7

Values are mean ± S.E.M.

There was no significant difference among the three groups in plasma TC, LDL-C, HDL-C, glucose and triglyceride.

Abbreviations: TC, total cholesterol; LDL-C, low-density lipoprotein cholesterol; HDL-C, high-density lipoprotein cholesterol

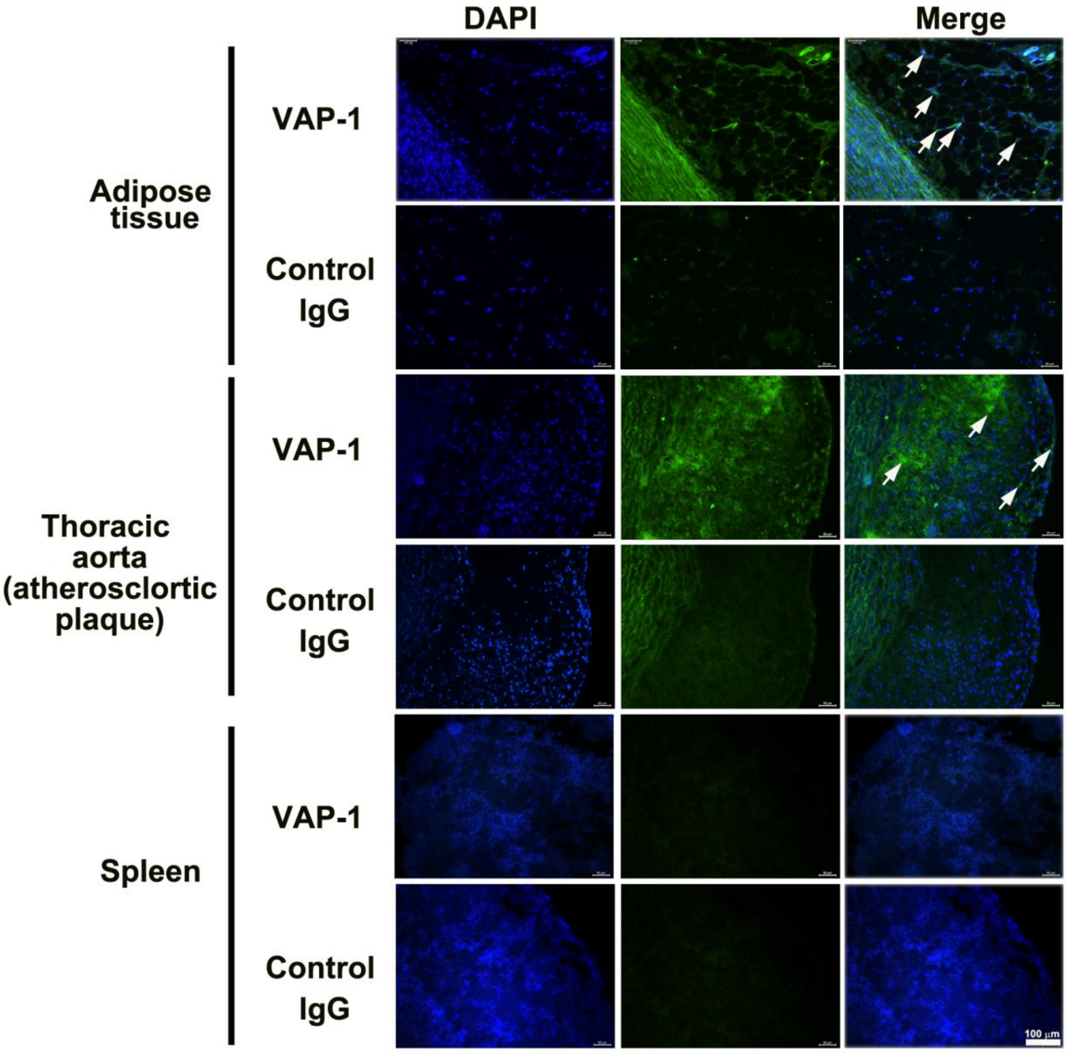

Supplementary Figure S1. Immunohistochemical staining with the antibodies against VAP-1 or control IgG in adipose tissue, thoracic aorta and spleen. VAP-1 stains (arrows) were found in adipose tissue and thoracic aorta (positive controls), but not in spleen (negative control).

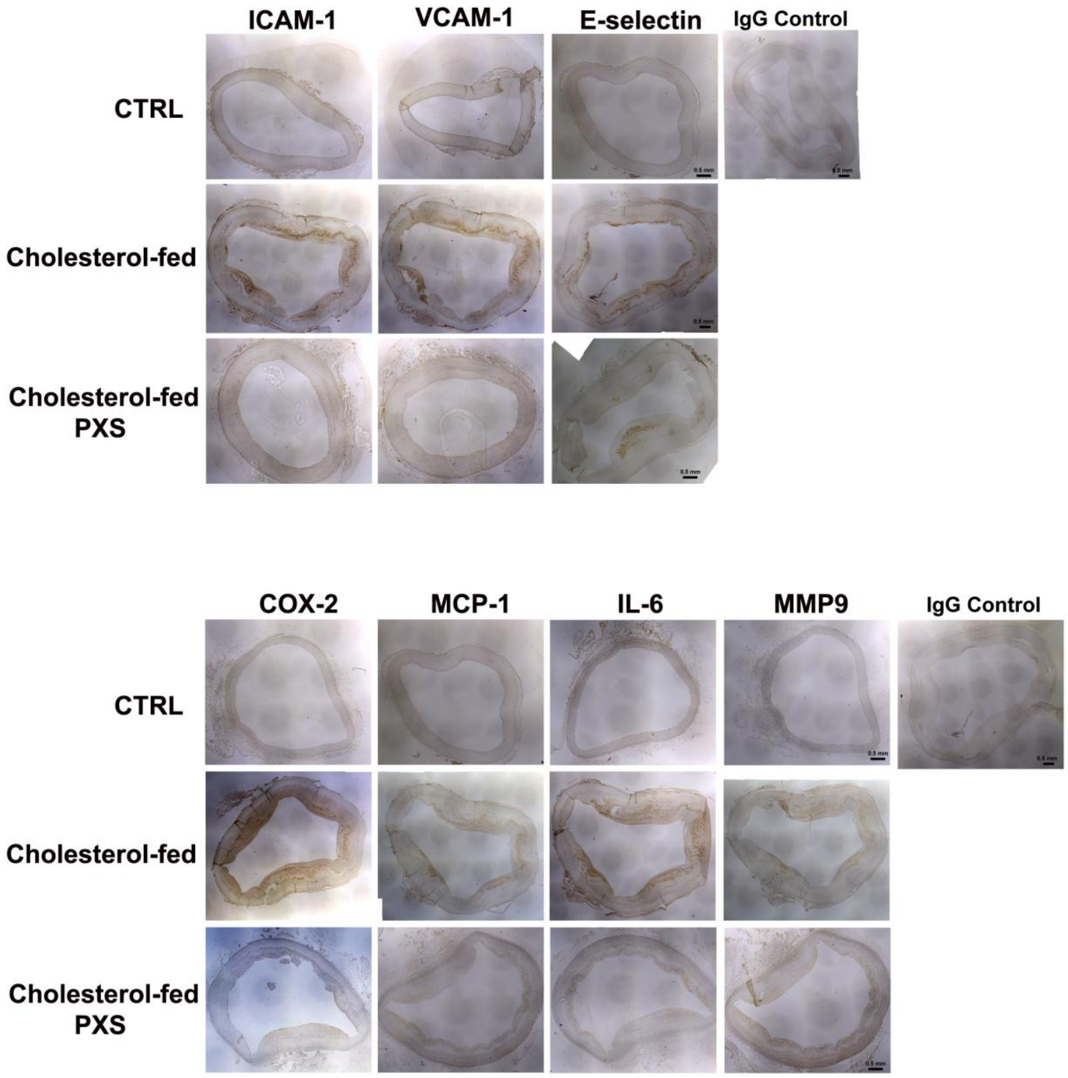

Supplementary Figure S2. Effects of semicarbazide-sensitive amine oxidase (SSAO) inhibition by PXS-4728A on the adhesion and inflammation in atherosclerotic plaques of thoracic aorta in rabbits in different groups. Results for the whole section of aorta in low power field of views (4X) were shown. Immunohistochemical staining for intercellular adhesion molecule-1 (ICAM-1), vascular cell adhesion molecule-1 (VCAM-1), E-selectin, cytokines including cyclooxygenase-2 (COX-2), monocyte chemoattractant protein-1 (MCP-1), and interleukin-6 (IL-6) and matrix metalloproteinase-9 (MMP9) were demonstrated.

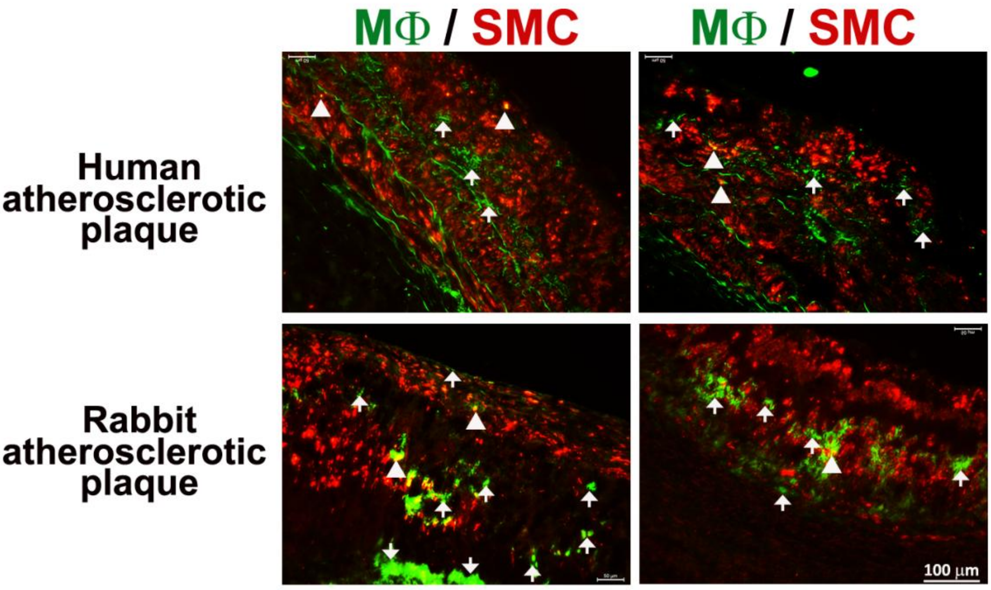

Supplementary Figure S3. Immunohistochemical staining with the antibodies against macrophage marker RAM11 (Mϕ, green fluorescence, arrows) and smooth muscle cell marker SMC (red fluorescence) by double immunofluorescent staining in human and rabbit atherosclerotic plaques. Only a small portion of macrophage activation markers were colocalized with SMC (arrowhead) in the atherosclerotic plaque.

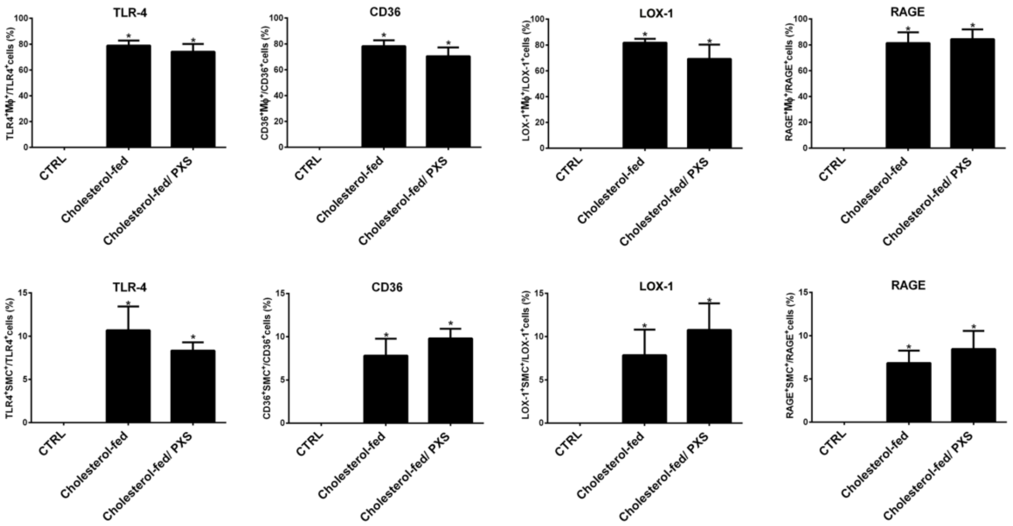

Supplementary Figure S4. Quantification of markers for macrophage activation and recruitment co-localized with markers for macrophage (Mϕ) and smooth muscle cells (SMC) in different groups. Immunohistochemical staining for Toll-like receptor -4 (TLR-4), CD36, lectin-like oxidized low-density lipoprotein receptor-1 (LOX-1), receptor for advanced glycation end-product (RAGE), macrophage (Mϕ) and smooth muscle cells (SMC) expression are shown. Most of TLR-4, CD36, LOX-1, and RAGE staining is closely co-localized with macrophages (M), and only a few co-localized with SMC.

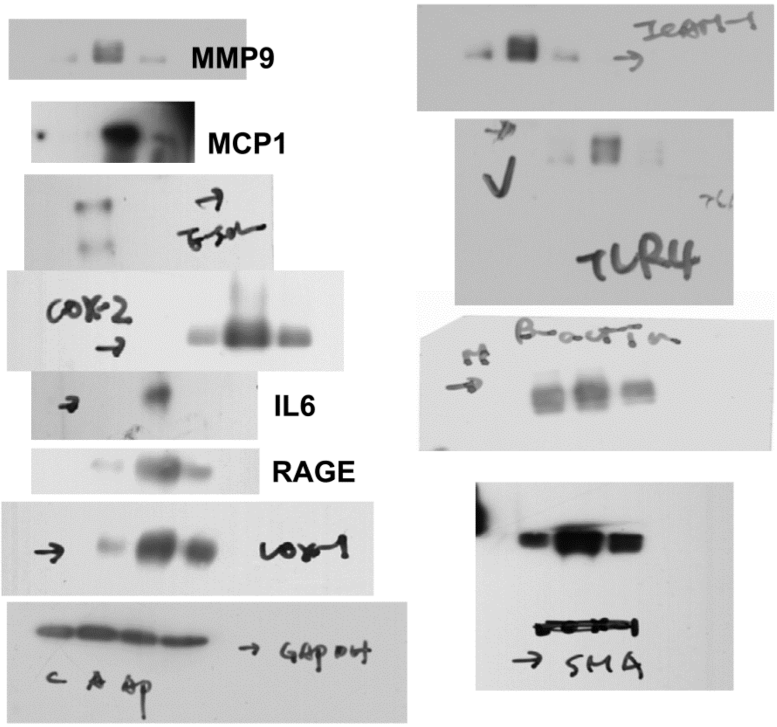

Supplementary Figure S5. Full length blots of Figure 4D. Black boxes show the cropping locations.
